# A Small Peptide with Therapeutic Potential for Inflammatory Acne Vulgaris

**DOI:** 10.1371/journal.pone.0072923

**Published:** 2013-08-28

**Authors:** Zhiye Zhang, Lixian Mu, Jing Tang, Zilei Duan, Fengyu Wang, Lin Wei, Mingqiang Rong, Ren Lai

**Affiliations:** 1 Key Laboratory of Animal Models and Human Disease Mechanisms of Chinese Academy of Sciences & Yunnan Province, Kunming Institute of Zoology, Kunming, Yunnan, China; 2 Life Sciences College of Nanjing Agricultural University, 1st Weigang, Nanjing, Jiangsu, China; 3 University of Chinese Academy of Sciences, Beijing, China; National Institutes of Health, United States of America

## Abstract

A designed peptide named LZ1 with 15 amino acid residues containing strong antimicrobial activity against bacteria pathogens of acne vulgaris including *Propionibacterium acnes*, *Staphylococcus epidermidis* and *S. aureus*. Especially, it exerted strong anti*-P. acnes* ability. The minimal inhibitory concentration against three strains of *P. acnes* was only 0.6 µg/ml, which is 4 times lower than that of clindamycin. In experimental mice skin colonization model, LZ1 significantly reduced the number of *P. acnes* colonized on the ear, *P. acnes*-induced ear swelling, and inflammatory cell infiltration. It ameliorated inflammation induced by *P. acnes* by inhibiting the secretion of inflammatory factors including tumor necrosis factor-α (TNF-α) and interleukin (IL)-1β. LZ1 showed little cytotoxicity on human keratinocyte and hemolytic activity on human blood red cells. Furthermore, LZ1 was very stable in human plasma. Combined with its potential bactericidal and anti-inflammatory properties, simple structure and high stability, LZ1 might be an ideal candidate for the treatment of acne.

## Introduction

Acne vulgaris is a common disorder with many physiological symptoms including comedones, papules, pustules, nodules, cysts and pilosebaceous inflammation [1], which are rarely life threatening, however, the greatest attention should be paid to the psychological morbidity [2]. Follicular colonisation by *P. acnes* plays a critical role in the development of inflammatory acne. Chemotactic factors induced by *P. acnes* attract neutrophils, monocytes, and lymphocytes to the pilosebaceous unit [3,4]. Furthermore, *P. acnes* induces initiation of sebum production in facial follicles [5,6], and stimulates the production of proinflammatory cytokines such as TNF-α, IL-1β, IL-8, and IL-12 mediated by toll-like receptor 2 [7–9]. In addition, *P. acnes* releases lipases, proteases, and hyaluronidases which contribute to tissue injury [10–13]. In response to *P. acnes*, keratinocytes can produce massive amounts of reactive oxygen species (ROS) by NAD(P)H oxidase through activation of the scavenger receptor CD36 to eliminate the bacteria and generate inflammation [14].

The ideal treatment for acne is the agent with potent antimicrobial and anti-inflammatory properties. Topical therapies including benzoyl peroxide (BPO), retinoids, and antibiotics, which are widely used for acne patients. BPO is one of the most frequently used epicutaneous medications to decrease *P. acnes* population for patients with mild-to-moderate facial acne [15]. The mechanism of BPO for acne treatment is thought to be antibacterial activity, but with little anti-inflammatory property [16]. In addition, the use of BPO is limited by its side-effects including erythema, scaling, burning, and flare [17]. Oral isotretinoin may be the most effective therapy and used early in severe disease due to its anti-inflammatory ability, but it not good for women of childbearing age because of the teratogenicity and other side-effects [2]. Antibiotics can improve acne, which seem to act directly on *P. acnes* and reduce inflammation. However, antibiotic resistance has been increasing in prevalence within the dermatologic setting [19], making it more and more difficult to cure acne. Long term antibacterial treatments may induce a gram-negative folliculitis (GNF) in patients with acne [20]. Thus, new agents containing both antimicrobial and anti-inflammatory activities and weak potential to induce drug-resistance are needed for acne treatment. The current study was performed to investigate the antimicrobial effects of LZ1 *in vitro* and *in vivo*. Moreover, we also sought to determine whether the peptide has therapeutic potential to treat acne.

## Materials and Methods

### Peptides synthesis

The peptide (LZ1, VKRWKKWWRKWKKWV-NH2) was synthesized by GL Biochem (Shanghai) Ltd. (Shanghai, China) and analyzed by reversed phase high performance liquid chromatography (RP-HPLC) and mass spectrometry to confirm it purity higher than 98%.

### Preparation of bacteria

Three strains of *P. acnes* (ATCC6919, ATCC 11827 and a clinically isolated strain with clindamycin-resistance), *S. epidermidis* (09A3726 and 09B2490) and *S. aureus* (ATCC 2592) were obtained from Kunming Medical College. The strains of *P. acnes* were cultured in brain heart infusion (BHI) broth (HKM, Guangzhou, China) with 1% glucose at 37°C under an anaerobic atmosphere using MGC Anaeropack systems (Mitsubishi, Gas Chemical Co., Inc, Japan), respectively. *P. acnes* grew to exponential-phase for 3 days and to stationary phase for 5 days in BHI broth [21]. *S. epidermidis* (09A3726 and 09B2490) and *S. aureus* (ATCC 2592) were grown in LB (Luria-Bertani) broth as our previous report [22].

### *In vitro* antimicrobial testing

MIC (minimal inhibitory concentration) of LZ1 and clindamycin phosphate, which has long been used clinically in acne treatment [23,24], and was used as control, was determined using broth dilution determination as our previous methods [21]. Briefly, samples were prepared as a stock solution in 0.9% salt water at a series of concentration. 890 µl special broth (BHI broth for *P. acnes*, LB broth for *S. epidermidis* and *S. aureus*), 100 µl bacterial suspension (10^8^ CFU/ml) and 10 µl test sample were put together in the test tube under anaerobic conditions at 37°C for 72 h for *P. acnes* and under aerobic conditions at 37°C for 24 or 48 h for *S. epidermidis* and *S. aureus*. An absorbance at 600 nm was measured by a microplate reader to estimate bacterial growth. The MIC was defined as the lowest concentration of test sample completely inhibiting microorganism’s growth.

### Assays of hemolysis and cytotoxicity

Hemolysis assay was undertaken using human red blood cells in liquid medium as our previous reports [22,25]. Serial dilutions of testing sample were incubated with human red cells at 37°C. After the incubation for 30 min, the cells were centrifuged and the absorbance in the supernatant was measured at 540 nm. Maximum hemolysis was determined by adding 1% Triton X-100 to a sample of cells. Hemolysis of testing samples was calculated as the percentage of cytotoxicity of Triton X-100.

Human HaCaT keratinocyte cells (1×10^5^ cells/ml, obtained from Cell Bank of Kunming Institute of Zoology, Chinese Academy of Sciences) were cultured in 96-well plates with Dulbecco’s modified Eagle’s medium (DMEM, Gibco) containing 10% fetal calf serum and penicillin (100 u/ml)-streptomycin (100 mg/ml) at 37°C in a humidified 5% CO_2_ atmosphere. Cell viability was evaluated by conventional 3-(4, 5-dimethyl-2-thiazolyl)-2, 5-diphenyl-2H-tetrazolium bromide (MTT) reduction assays. After a 24-h treatment by testing sample, 20 µl of MTT (5 mg/ml) was added to each well. The MTT solution was then removed and 200 µl dimethyl sulfoxide (DMSO) was added to solubilize the MTT-formazan cristals in living cells. The absorbance at 570 nm of the resulting solution was measured. The experiments were performed in triplicate.

### Effects of human plasma on LZ1’s antibacterial activity

LZ1 or human cathelicidin antimicrobial peptide LL-37 (control) was mixed with human plasma (final concentration 100 µM) for 0, 0.5, 1, 2, 4, 6 and 8 hours at 37°C, respectively. Residual antibacterial activity was evaluated by using 10 µl aliquot of the mixture to measure inhibitory zone on bacterial growth on BHI or LB broth containing 0.7% agar as previously described [26]. The diameters of inhibition zones were recorded after 72-hour incubation for *P. acnes* and 24-hour incubation for *S. epidermidis* and *S. aureus*, respectively.

### *In vivo anti-P. acnes* experiments

As described previously [1,21], *P. acnes* (ATCC6919) was grown to the exponential-phase in BHI broth and then centrifuged at 1000 g for 10 min. The bacterium pellet was then washed twice and re-suspended in 0.9% salt water (5×10^8^ CFU/ml). Kunming mice (20 ± 2 g) were anesthetised by intraperitoneal (IP) administration of ketamine (50 mg/kg) and xylazine (15 mg/kg), and then 20 µl *P. acnes* solution was intradermally injected into left ears of the mice. Right ears received the same volume of 0.9% salt water. Placebo or 0.2% LZ1 gel (Polyethylene Glycol (PEG) 400: PEG 4000, 1:1) was applied on the surface of right ear skin after injection with *P. acnes* or saline. 0.2% clindamycin gel or vehicle was applied on the ear skin surface of mice as the control. Ear thickness after 24 h bacterial injection was measured using a micro caliper.

To determine *P. acnes* number in the ear, the left ear was cut off after 24 h bacterial injection and the mice were sacrificed immediately by cervical dislocation. The ears were wiped to remove gel and then homogenized in 0.9% salt water (1 ml per ear) with a hand tissue grinder. CFUs of *P. acnes* in the ear were enumerated by plating serial dilutions of the homogenate on BHI plates and the bacterial numbers were counted after 72 h incubation under anaerobic conditions at 37°C.

To further investigate the therapeutical effect, the increase in ear thickness and the *P. acnes* number in the ear after bacterial injection were determined continuously on day 2 to day 5 as described above. LZ1, clindamycin or vehicle was applied on the ear skin surface of mice once per day. All the experimental protocols to use animals were approved by the Animal Care and Use Committee at Kunming Institute of Zoology, Chinese Academy of Sciences.

### Cytokine measurement

Ears of Kunming mice were injected with live *P. acnes* (ATCC 6919) as described above. The ear was excised on day 1 to day 5 after bacterial injection respectively, and then homogenized in 0.9% salt water (1 ml per ear) with a hand tissue grinder. The supernatant was collected after the centrifugation at 2000 × g for 10 min and then was used for cytokine measurement. The concentration of TNF-α and IL-1β was measured by using enzyme-linked immunosorbent assay kits (Excell Bio, Shanghai, China).

### Histological check

Ears of Kunming mice were excised and fixed in neutral-buffered 10% formalin solution and embedded in paraffin by routine procedures. The slides were cut at approximately 4 µm and stained routinely with haematoxylin and eosin (HE). Images were obtained using a Nikon Eclipse 80i microscope (Nikon, Japan).

### Statistical analysis

Data were assessed for statistical significance using Student’s (unpaired) *t*-test. Results were reported as mean ± SE with significance accepted at *P*< 0.05.

## Results

### Antimicrobial activities of LZ1

As listed in table 1, peptide LZ1 showed strong antimicrobial activities against tested microorganisms. Three *P. acnes* strains including a clinically isolated strain with clindamycin resistance were used in this experiment. LZ1 showed the same antimicrobial activity against these three strains. The MIC value was 0.6 µg/ml. Clindamycin showed antimicrobial activity against two of them with a MIC of 2.3 µg/ml. This suggests that LZ1 has stronger anti*-P. acnes* activity than clindamycin *in vitro*. LZ1 also showed antimicrobial activity (with a MIC of 4.7 µg/ml) against *S. epidermidis* 09A3726, but clindamycin had no activity against it. For *S. epidermidis* 09B2490 and *S. aureus* 09B2499, the MIC value of the peptide was 2.3 µg/ml, while that of clindamycin was 1.2 µg/ml.

**Table 1:** Antimicrobial activities of LZ1 against skin bacteria.

	**MIC (μg/ml)**	
**Bacteria**	**LZ1**	**CL**
*P. acnes* ATCC6919	0.6	2.3
*P. acnes* ATCC11827	0.6	2.3
*P. acnes* (IS, DR)	0.6	ND
*S. epidermidis* 09A3726	4.7	ND
*S. epidermidis* 09B2490	2.3	1.2
*S. aureus* 09B2499	2.3	1.2

MIC: minimal peptide concentration required for total inhibition of cell growth in liquid medium. These concentrations represent mean values of three independent experiments performed in duplicates. IS: clinically isolated strain, DR: drug resistance for clindamycin, CL: clindamycin, ND: no detectable activity

### Cytotoxic and hemolytic assays

**Figure 1:**
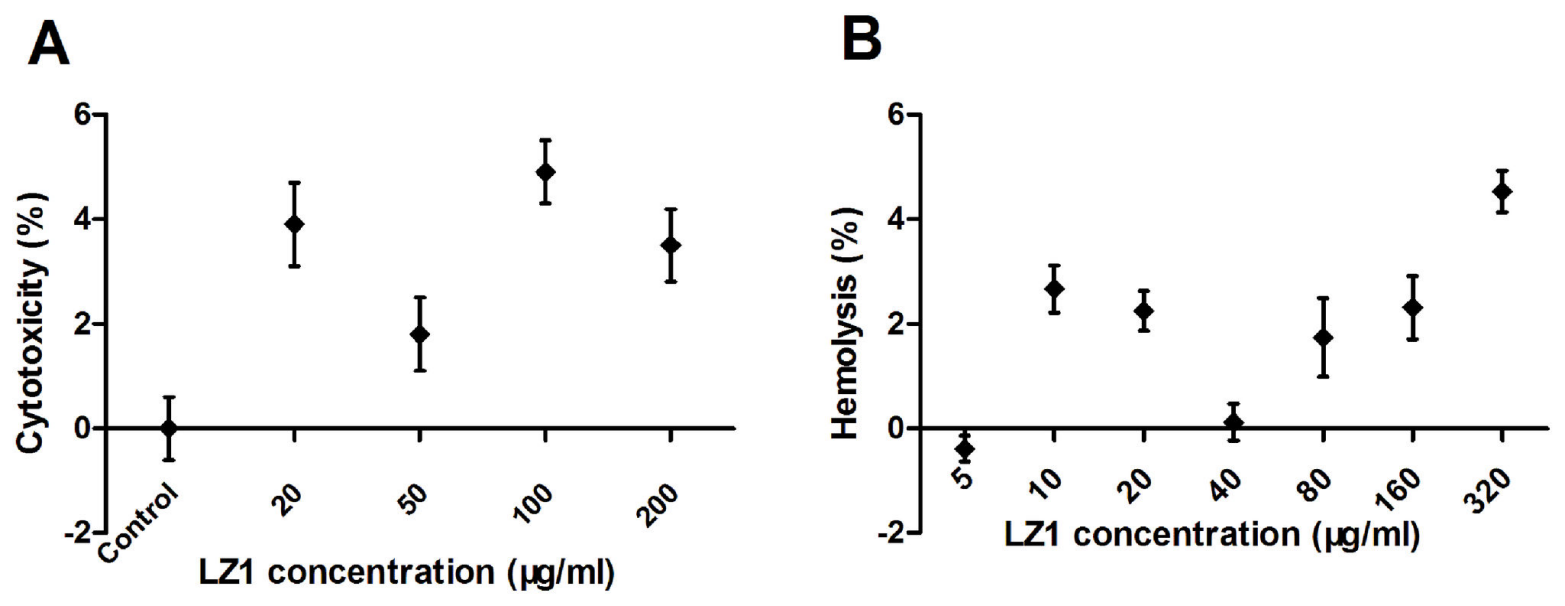
Cytotoxicity and hemolysis of LZ1. (A) Cytotoxicity of LZ1 on human keratinocytes. (B) Hemolysis of LZ1 on human red blood cells. Data represent mean ± SE of five individual experiments.

Human keratinocytes are major cells that *P. acnes* interacts with, and this interaction induces the release of inflammatory cytokines [28–30]. Human HaCaT keratinocyte cells were used to evaluate LZ1’s cytotoxicity. As illustrated in Figure 1A, HaCaT keratinocyte cells were incubated with LZ1 at various concentrations from 20 to 200 µg/ml. LZ1 exerted little effects on cell viabilities (no more than 5.6%). In addition, LZ1 showed little hemolytic activity (no more than 5.2%) on human red blood cells even with a peptide concentration up to 320 µg/ml as illustrated in Figure 1B.

### The effect of human plasma on the antibacterial activity of LZ1

**Figure 2:**
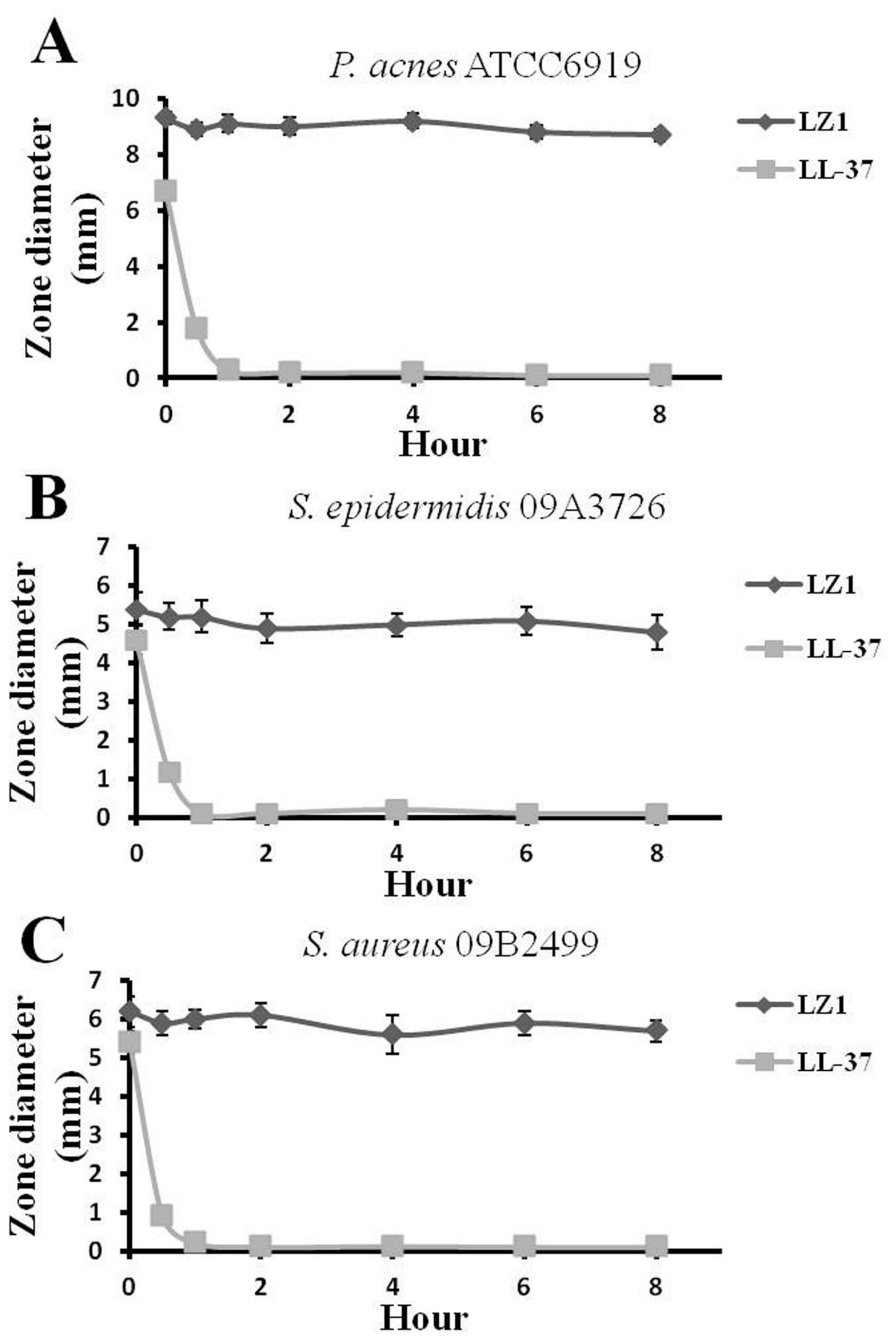
Effects of human plasma on the antibacterial activity of LZ1 and LL-37 against *P. acnes* ATCC6919 (A), *S. epidermidis* 09A3726 (B) and *S. aureus* 09B2499 (C). Data represent mean ± SE of three individual experiments.

The effect of human plasma on the antibacterial activity of LZ1 was investigated. As illustrated in Figure 2, the antibacterial activity of LZ1 on tested three microorganisms could not be significantly affected by the incubation with human plasma even for 8 hours while human cathelicidin antimicrobial peptide LL-37, completely lost its antibacterial activity after 1 hour incubation with human plasma at 37°C.

### Effects of LZ1 on *P. acnes* -induced inflammation *in vivo*

**Figure 3:**
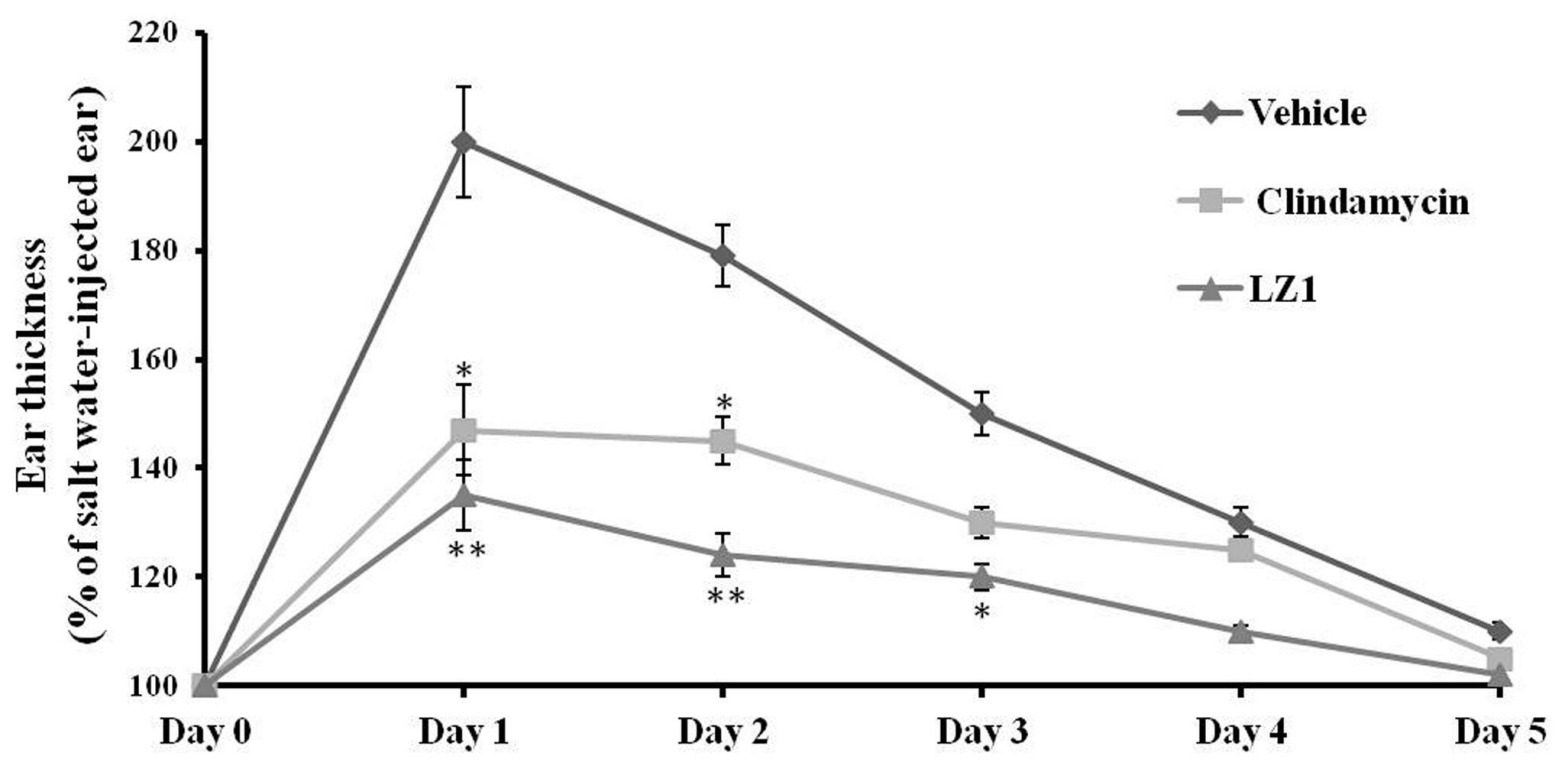
Effects of LZ1 and clindamycin epicutaneous application on *P. acnes*-induced ear swelling. The increase in ear thickness of the left ear was calculated as percentage of the right ear. Data represent mean ± SE of four individual experiments. The values for LZ1- or clindamycin-treated group were significant different from the value for the vehicle group (*P< 0.05, **P< 0.001).

Ear swelling induced by *P. acnes* challenge was observed as illustrated in Figure 3. After one-day *P. acnes* challenge, the ear thickness of vehicle mice was two times of that of un-challenged mice (the control). The corresponding ear thickness for LZ1 and clindamycin-treated mice was 135% and 147% of that of the control, respectively. No swelling was observed in control mice ear injected only with saline. All mice were recovered gradually. At day 2 to day 5, the ear thickness of vehicle mice was 179%, 150%, 130%, and 110% of that of the control while that of LZ1-treated group was 124%, 120%, 110%, and 102% of that of the control, respectively. For the positive group (clindamycin-treated), the ear thickness was 145%, 130%, 125%, and 105% of that of the control, respectively.

**Figure 4:**
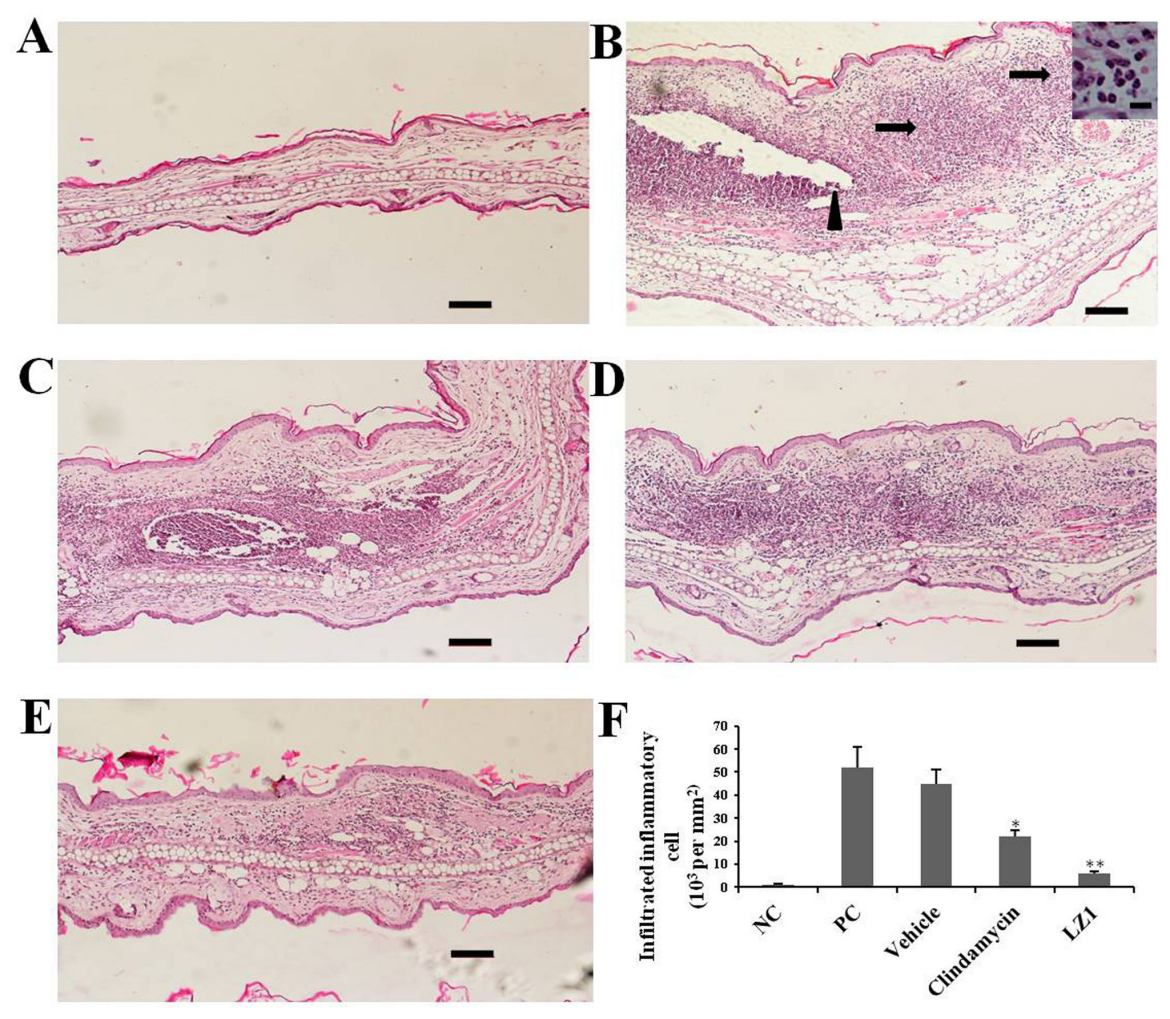
Histopathological analysis of mouse ears. (A) Mouse ear injected with only 0.9% salt water. (B) After 24 h *P. acnes* challenge, ear swelling and the infiltrated inflammatory cells (arrows) surrounding the injection site of *P. acnes* (arrowhead) were observed. Image of inflammatory cells at higher magnification was shown in subsets, scale bar=10µm. (C) Ear swelling has not been changed by vehicle application. Clindamycin (D) and LZ1 application (E) effectively ameliorated ear swelling. (F) The number of infiltrated inflammatory cells was counted (*P< 0.05, **P< 0.001). Data are representative of four separate experiments with similar results. Scale bar =100µm. NC: no challenge by *P. acnes*; PC: mice subjected to *P. acnes* challenge for 24 h without any treatment.

The anti-inflammatory effect of LZ1 was further evaluated by histological check (Figure 4). Intradermally injected *P. acnes* induced significant ear inflammation and swelling in the ear of experimental mice as illustrated in Figure 4B. Many infiltrated inflammatory cells could be found in the injection site of *P. acnes*. After epicutaneous application of 0.2% LZ1 gel on the mice ear for 24 h, the inflammation induced by *P. acnes* was ameliorated effectively (Figure 4E), and the number of infiltrated inflammatory cells decreased markedly (Figure 4F). In contrast, ear swelling could not be reduced by epicutaneously application of vehicle (Figure 4C). The positive control clindamycin also effectively inhibited ear inflammation induced by *P. acnes* injection (Figure 4D) and decreased infiltrated inflammatory cells (Figure 4F), however, the therapeutic potential of clindamycin was not as good as that of LZ1.

**Figure 5:**
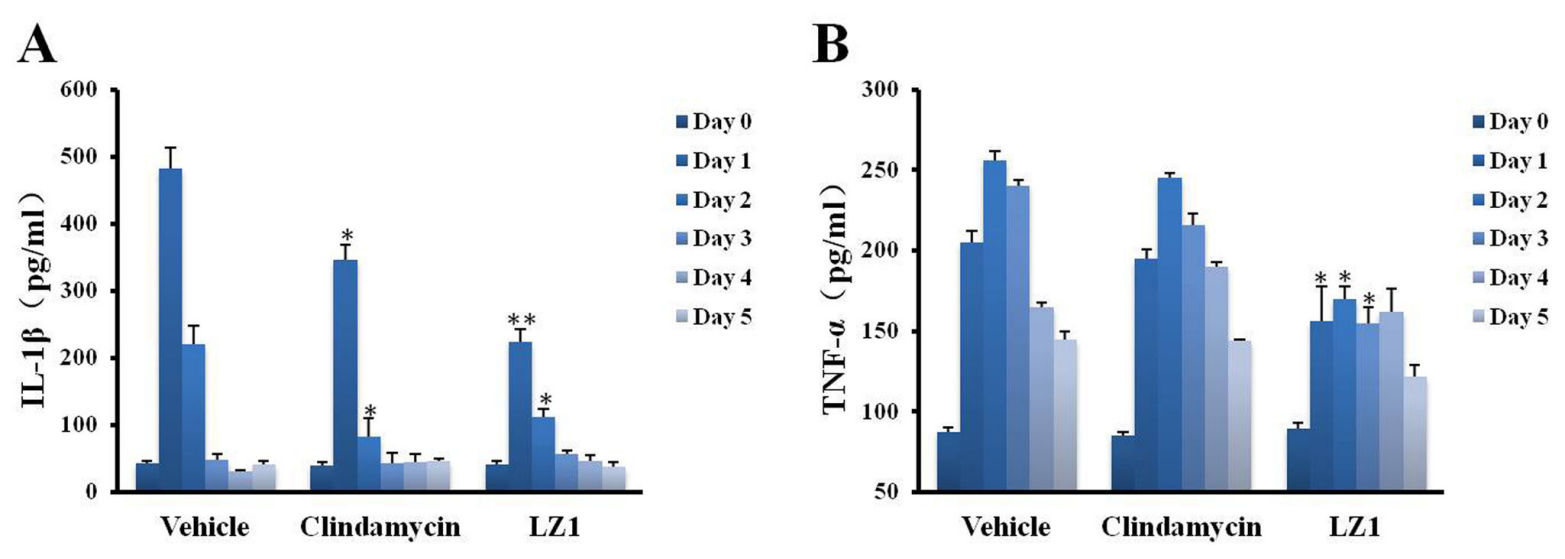
Effects of LZ1 on cytokine production induced by *P. acnes*. (A) the effect of LZ1 and clindamycin on IL-1β production induced by *P. acnes*. (B) the effect of LZ1 and clindamycin on TNF-α production induced by *P. acnes*. Data represent mean ± SE of three individual experiments. The values for LZ1- or clindamycin-treated group were significant different from the value for the vehicle group (*P< 0.05, **P< 0.001).

Inflammatory acne was regulated by inflammatory factors including TNF-α and IL-1β, which are secreted by monocytes [7]. Infiltrated inflammatory cells were attracted by these inflammatory factors induced by *P. acnes* [9]. The level of inflammation factor represents inflammation degree. These two cytokines were monitored during the epicutaneously application of tested drugs on the inflammatory acne model. As illustrated in Figure 5, the secretion of both TNF-α and IL-1β were markedly increased by *P. acnes* challenge. The level of IL-1β and TNF-α was 40 and 80 pg/ml in normal mouse while the level was increased to 483 and 205 pg/ml after one-day *P. acnes* challenge, respectively. LZ1 and clindamycin significantly inhibited IL-1β secretion induced by *P. acnes*. IL-1β concentration in mouse ear (1 ml saline per ear) was 223, 111, 57, 47, 38 pg/ml at day 1 to day 5 after LZ1 application while IL-1β concentration in vehicle group was 483, 221, 48, 30, 41 pg/ml respectively. The corresponding IL-1β concentration in clindamycin-treated mice was 346, 83, 43, 45, 46 pg/ml, respectively (Figure 5A). LZ1 also inhibited TNF-α secretion induced by *P. acnes* challenge while clindamycin had no inhibitory effect on it. Compared with TNF-α concentration of 205, 256, 240, 165, 145 pg/ml at day 1 to day 5 after *P. acnes* challenge in the vehicle group, the TNF-α value was decreased to 156, 170, 155, 162, 122 pg/ml after the epicutaneously application of LZ1, respectively (Figure 5B).

### LZ1’ inhibition on *P. acnes* conlonization *in vivo*

The number of *P. acnes* colonized within the ear was reduced noticeably by LZ1 application (Table 2). The number of *P. acnes* in the vehicle group at day 1 to day 5 was 8.4 ×10^5^, 7.8 × 10^4^, 3.4 × 10^4^, 2.1 × 10^4^, 3 × 10^3^ CFU per ear, respectively. The number of *P. acnes* colonized within the ear was reduced gradually, but both LZ1 and clindamycin significantly accelerated elimination of the *P. acnes*. LZ1 treatment led to 75.1, 62.8, 70.6, 76.2 and 56.7% reduction in the number of *P. acnes* colonized within the ear at day 1 to day 5, respectively. Clindamycin treatment led to 57.1, 58.9, 58.8, 71.4 and 50% reduction of *P. acnes* at day 1 to day 5, respectively.

## Discussion

Looking back over the past 50 years, topical and oral antibiotics have been the most effective therapy for acne treatment. Antibiotics demonstrate antimicrobial effect and inhibit the production of *P. acnes*-associated inflammatory factors. However, the increasing emergence of resistant *P. acnes* strains is causing worldwide concern. There was evidence of antibiotic-resistant *P. acnes* emerged after only 8 weeks of topical antibiotic monotherapy [18]. Antibiotic resistant and multiple drug resistant *P. acnes* strains have been identified from acne patients with long-term antibiotic treatment [31,32]. Evidences suggest that the use of topical erythromycin and clindamycin leads to the gradual increase in drug resistance [33–35]. The problem of antibiotic resistance causes the failure of antibiotic treatment for acne. Furthermore, the resistant genes may transfer to other potentially pathogenic bacteria, such as certain strains of *staphylococci* or *streptococci* [36,37]. Great efforts aim to limit antibiotic resistance in acne, including necessary addition of benzoyl peroxide (BPO) and retinoid. Nevertheless, to identify new therapeutic agents without inducing resistance will be another good way in acne.

**Table 2:** The number of *P. acnes* colonized within the ear after epicutaneous application of LZ1.

	***P. acnes* (10^3^ CFU per ear)**				
	Day 1	Day 2	Day 3	Day 4	Day 5
Vehicle	844±89	78±8.2	34±2.0	21±1.8	3±0.3
Clindamycin	360±77	32±4.1	14±1.7	6±0.5	1.5±0.1
LZ1	210±34	29±5.4	10±1.5	5±0.4	1.3±0.1

Data represent mean of four individual experiments.

Antimicrobial peptides (AMPs) are the first line of innate immunity against invading microorganisms and play roles in the control of the natural microbial flora. The protective functions provided by AMPs in external skin surface were understood until recently. For example, the human β-defensin family has been found in human pilosebaceous units, which may be involved in the pathogenesis of acne vulgaris [38]. hCAP18/LL-37 containing ability to kill *P. acnes* was found in sebaceous glands [39]. More importantly, AMPs have been demonstrated that they have low potential to induce drug resistance of microorganisms [40,41]. Among the AMPs, there has been increasing interest in a specific subset of them – the tryptophan (Trp), lysine (Lys) or arginine (Arg) rich peptides [42–45]. These residues possess some specific chemical properties that make them suitable for antimicrobial peptides. Studies focused on these peptides facilitated the understanding of the molecular mechanisms of the AMPs. The hydrophobic Trp has preference for the interfacial region of lipid bilayers, while Lys and Arg residues endow the peptides with cationic charges and hydrogen bonding properties crucial for interaction with the abundant anionic components of the bacterial membrane [46,47]

In this study, a Trp–Lys-rich peptide LZ1 was screened and its antimicrobial potential was evaluated. This peptide has a linear primary structure and it just composed of 15 amino acid residues. It showed strong antimicrobial abilities against bacteria pathogens of acne vulgaris, such as *P. acnes*, *S.* epidermidis and *S. aureus in vitro* (Table 1). The corresponding MIC was 0.6 to 4.7 µg/ml. It exerted the same antimicrobial abilities against both common and antibiotic resistant bacterial strains (Table 1). The *in vivo* anti-*P. acnes* ability of LZ1 was also investigated in mouse skin colonization model. The clearance of *P. acnes* colonized on the mouse ear was accelerated by LZ1 (Table 2).

Some studies have shown that many cationic antimicrobial peptide exerted hemolytic abilities on human blood red cells [27,28,48,49]. Two possible side effects including hemolysis and cytotoxicity exerted by some AMPs were tested. LZ1 showed little hemolytic activity and cytotoxicity even at a high concentration (>200 µg/ml, Figure 1). A drug may be modified or degraded due to various proteases in plasma, which is the most important problem in drug application. This peptide seems to be very stable in human plasma since its antimicrobial activity was not lost even LZ1’s incubation with human plasma for 8 h at 37°C. Binding to apolipoprotein A-I (apoA-I) and glycosaminoglycan inhibits the antibacterial activity of LL-37 [50,51].

Another significant feature of LZ1 is that it exerted strong anti-inflammatory effects. Follicular colonisation by *P. acnes* plays an important role in the formation of acne. The proliferation of *P. acnes* will attract CD4+ lymphocytes and macrophages to microcomedone [52] and then induce the inflammatory acne lesion with rupture of follicular wall. As illustrated by Figure 4B, injection of *P. acnes* attracted plenty of infiltrated inflammatory cells. After epicutaneous administration of LZ1, infiltrated inflammatory cells were decreased markedly (Figure 4E, F), and ear swelling induced by *P. acnes* was inhibited significantly (Figure 3), suggesting a strong anti-inflammatory effect. After the treatment for acne with LZ1 for 5 days, two important inflammatory cytokines including TNF-α and IL-1β induced by *P. acnes* were significantly inhibited by the peptide (Figure 5A, B), suggesting epicutaneous administration of LZ1 suppressed the inflammation in acne though inhibiting the production of inflammatory cytokines.

In conclusion, we demonstrated the antimicrobial effect of LZ1 against skin bacteria *in vitro* and its therapeutic potential for inflammatory acne vulgaris induced by *P. acnes in vivo* using mouse ear model. Combined its simple primary structure with only 15 amino acid residues, which facilitates to produce, ship and store, little hemolytic activity on blood red cells, little cytotoxicity on human keratinocytes, and high stability in human plasma, LZ1 might be an excellent therapeutic agent for the treatment of acne vulgaris although much more work is necessary.

## References

[1] Nakatsuji T, Kao MC, Fang JY, Zouboulis CC, Zhang L et al. (2009) Antimicrobial property of lauric acid against *Propionibacterium acnes*: its therapeutic potential for inflammatory acne vulgaris. J Invest Dermatol 129: 2480-2488. doi:10.1038/jid.2009.93. PubMed: 19387482. 19387482 10.1038/jid.2009.93 PMC2772209

[2] Williams HC, Dellavalle RP, Garner S (2012) Acne vulgaris. Lancet 379: 361-372. doi:10.1016/S0140-6736(11)60321-8. PubMed: 21880356. 21880356 10.1016/S0140-6736(11)60321-8

[3] Webster GF, Leyden JJ (1980) Characterization of serum-independent polymorphonuclear leukocyte chemotactic factors produced by *Propionibacterium acnes* . Inflammation 4: 261-269. doi:10.1007/BF00915027. PubMed: 7429606. 7429606 10.1007/BF00915027

[4] Burkhart CG, Burkhart CN, Lehmann PF (1999) Acne: a review of immunologic and microbiologic factors. Postgrad Med J 75: 328-331. PubMed: 10435165. 10435165 10.1136/pgmj.75.884.328 PMC1741272

[5] Mourelatos K, Eady EA, Cunliffe WJ, Clark SM, Cove JH (2007) Temporal changes in sebum excretion and propionibacterial colonization in preadolescent children with and without acne. Br J Dermatol 156: 22-31. doi:10.1111/j.1365-2133.2006.07517.x. PubMed: 17199562. 17199562 10.1111/j.1365-2133.2006.07517.x

[6] Iinuma K, Sato T, Akimoto N, Noguchi N, Sasatsu M et al. (2009) Involvement of *Propionibacterium acnes* in the augmentation of lipogenesis in hamster sebaceous glands in vivo and in vitro. J Invest Dermatol 129: 2113-2119. doi:10.1038/jid.2009.46. PubMed: 19282842. 19282842 10.1038/jid.2009.46

[7] Vowels BR, Yang S, Leyden JJ (1995) Induction of proinflammatory cytokines by a soluble factor of *Propionibacterium acnes*: implications for chronic inflammatory acne. Infect Immun 63: 3158–3165. PubMed: 7542639. 7542639 10.1128/iai.63.8.3158-3165.1995 PMC173431

[8] Nagy I, Pivarcsi A, Kis K, Koreck A, Bodai L et al. (2006) *Propionibacterium acnes* and lipopolysaccharide induce the expression of antimicrobial peptides and proinflammatory cytokines/chemokines in human sebocytes. Microbes Infect 8: 2195-2205. doi:10.1016/j.micinf.2006.04.001. PubMed: 16797202. 16797202 10.1016/j.micinf.2006.04.001

[9] Kim J, Ochoa MT, Krutzik SR, Takeuchi O, Uematsu S et al. (2002) Activation of toll-like receptor 2 in acne triggers inflammatory cytokine responses. J Immunol 169: 1535-1541. PubMed: 12133981. 12133981 10.4049/jimmunol.169.3.1535 PMC4636337

[10] Hoeffler U (1977) Enzymatic and hemolytic properties of *Propionibacterium acnes* and related bacteria. J Clin Microbiol 6: 555-558. PubMed: 201661. 201661 10.1128/jcm.6.6.555-558.1977 PMC274823

[11] Hoeffler U, Ko HL, Pulverer G (1976) Antimicrobiol susceptibility of *Propionibacterium acnes* and related microbial species. Antimicrob Agents Chemother 10: 387-394. doi:10.1128/AAC.10.3.387. PubMed: 984781. 984781 10.1128/aac.10.3.387 PMC429758

[12] Ingham E, Holland KT, Gowland G, Cunliffe WJ (1980) Purification and partial characterization of an acid phosphatase (EC 3.1.3.2) produced by *Propionibacterium acnes* . J Gen Microbiol 118: 59-65. PubMed: 7420057. 7420057 10.1099/00221287-118-1-59

[13] Puhvel SM, Reisner RM (1972) The production of hyaluronidase (hyaluronate lyase) by *Corynebacterium acnes* . J Invest Dermatol 58: 66-70. doi:10.1111/1523-1747.ep12551495. PubMed: 4258461. 4258461 10.1111/1523-1747.ep12551495

[14] Grange PA, Chéreau C, Raingeaud J, Nicco C, Weill B et al. (2009) Production of superoxide anions by keratinocytes initiates P. acnes-induced inflammation of the skin. PLOS Pathog 5: e1000527 PubMed: 19629174. 19629174 10.1371/journal.ppat.1000527 PMC2709429

[15] Ozolins M, Eady EA, Avery AJ, Cunliffe WJ, Po AL et al. (2004) Comparison of five antimicrobial regimens for treatment of mild to moderate inflammatory facial acne vulgaris in the community: randomized controlled trial. Lancet 364: 2188–2195. doi:10.1016/S0140-6736(04)17591-0. PubMed: 15610805. 15610805 10.1016/S0140-6736(04)17591-0

[16] Gollnick H, Schramm M (1998) Topical drug treatment in acne. Dermatology 196: 119–125. doi:10.1159/000017844. PubMed: 9557245. 9557245 10.1159/000017844

[17] Castro GA, Ferreira LA (2008) Novel vesicular and particulate drug delivery systems for topical treatment of acne. Expert Opin Drug Deliv 5: 665–679. doi:10.1517/17425247.5.6.665. PubMed: 18532922. 18532922 10.1517/17425247.5.6.665

[18] Cunliffe WJ, Holland KT, Bojar R, Levy SF (2002) A randomized, double-blind comparison of a clindamycin phosphate/benzoyl peroxide gel formulation and a matching clindamycin gel with respect to microbiologic activity and clinical efficacy in the topical treatment of acne vulgaris. Clin Ther 24: 1117-1133. doi:10.1016/S0149-2918(02)80023-6. PubMed: 12182256. 12182256 10.1016/s0149-2918(02)80023-6

[19] Swanson JK (2003) Antibiotic resistance of *Propionibacterium acnes* in acne vulgaris. Dermatol Nurs 15: 359–362. PubMed: 14515616. 14515616

[20] Böni R, Nehrhoff B (2003) Treatment of gram-negative folliculitis in patients with acne. Am J Clin Dermatol 4: 273-276. doi:10.2165/00128071-200304040-00005. PubMed: 12680804. 12680804 10.2165/00128071-200304040-00005

[21] Wang Y, Zhang Z, Chen L, Guang H, Li Z et al. (2011) Cathelicidin-BF, a snake cathelicidin-derived antimicrobial peptide, could be an excellent therapeutic agent for acne vulgaris. PLOS ONE 6: e22120. doi:10.1371/journal.pone.0022120. PubMed: 21789223. 21789223 10.1371/journal.pone.0022120 PMC3137605

[22] Li J, Xu X, Xu C, Zhou W, Zhang K et al. (2007) Anti-infection peptidomics of amphibian skin. Mol Cell Proteomics 6: 882–894. doi:10.1074/mcp.M600334-MCP200. PubMed: 17272268. 17272268 10.1074/mcp.M600334-MCP200

[23] Eichenfield LF, Wortzman M (2009) A novel gel formulation of 0.25% tretinoin and 1.2% clindamycin phosphate: efficacy in acne vulgaris patients aged 12 to 18 years. Pediatr Dermatol 26: 257-261. doi:10.1111/j.1525-1470.2008.00862.x. PubMed: 19706084. 19706084 10.1111/j.1525-1470.2008.00862.x

[24] Abdel-Naser MB, Zouboulis CC (2008) Clindamycin phosphate/tretinoin gel formulation in the treatment of acne vulgaris. Expert Opin Pharmacother 9: 2931-2937. doi:10.1517/14656566.9.16.2931. PubMed: 18937624. 18937624 10.1517/14656566.9.16.2931

[25] Lu Y, Ma Y, Wang X, Liang J, Zhang C et al. (2008) The first antimicrobial peptide from sea amphibian. Mol Immunol 45: 678–681. doi:10.1016/j.molimm.2007.07.004. PubMed: 17707909. 17707909 10.1016/j.molimm.2007.07.004

[26] Lai R, Zheng YT, Shen JH, Liu GJ, Liu H et al. (2002) Antimicrobial peptides from skin secretions of Chinese red belly toad *Bombina maxima* . Peptides 23: 427-435. doi:10.1016/S0196-9781(01)00641-6. PubMed: 11835991. 11835991 10.1016/s0196-9781(01)00641-6

[27] Yin LM, Edwards MA, Li J, Yip CM, Deber CM (2012) Roles of hydrophobicity and charge distribution of cationic antimicrobial peptides in peptide-membrane interactions. J Biol Chem 287: 7738-7745. doi:10.1074/jbc.M111.303602. PubMed: 22253439. 22253439 10.1074/jbc.M111.303602 PMC3293554

[28] Hwang H, Hyun S, Kim Y, Yu J (2013) Reduction of helical content by insertion of a disulfide bond leads to an antimicrobial peptide with decreased hemolytic activity. Chem Med Chem 8: 59-62. PubMed: 23150226. 23150226 10.1002/cmdc.201200463

[29] Graham GM, Farrar MD, Cruse-Sawyer JE, Holland KT, Ingham E (2004) Proinflammatory cytokine production by human keratinocytes stimulated with *Propionibacterium acnes* and *P. acnes* GroEL. Br J Dermatol 150: 421–428. doi:10.1046/j.1365-2133.2004.05762.x. PubMed: 15030323. 15030323 10.1046/j.1365-2133.2004.05762.x

[30] Schaller M, Loewenstein M, Borelli C, Jacob K, Vogeser M et al. (2005) Induction of a chemoattractive proinflammatory cytokine response after stimulation of keratinocytes with *Propionibacterium acnes* and coproporphyrin III. Br J Dermatol 153: 66–71. doi:10.1111/j.1365-2133.2005.06530.x. PubMed: 16029328. 16029328 10.1111/j.1365-2133.2005.06530.x

[31] Eady EA, Gloor M, Leyden JJ (2003) *Propionibacterium acnes* resistance: a worldwide problem. Dermatology 206: 54-56. doi:10.1159/000067822. PubMed: 12566805. 12566805 10.1159/000067822

[32] Nord CE, Oprica C (2006) Antibiotic resistance in *Propionibacterium acnes*. Microbiological and clinical aspects. Anaerobe 12: 207-210. doi:10.1016/j.anaerobe.2006.08.001. PubMed: 17000123. 17000123 10.1016/j.anaerobe.2006.08.001

[33] Eady EA, Cove JH, Holland KT, Cunliffe WJ (1989) Erythromycin resistant propionibacteria in antibiotic treated acne patients: association with therapeutic failure. Br J Dermatol 121: 51-57. doi:10.1111/j.1365-2133.1989.tb05952.x. PubMed: 2527056. 2527056 10.1111/j.1365-2133.1989.tb01399.x

[34] Gollnick H, Cunliffe W, Berson D, Dreno B, Finlay A et al. (2003) Management of acne: a report from a Global Alliance to Improve Outcomes in Acne. J Am Acad Dermatol 49: S1-37. 12833004 10.1067/mjd.2003.618

[35] Leyden JJ, Preston N, Osborn C, Gottschalk RW (2011) In-vivo Effectiveness of Adapalene 0.1%/Benzoyl Peroxide 2.5% Gel on Antibiotic-sensitive and Resistant *Propionibacterium acnes* . J Clin Aesthet Dermatol 4: 22-26. PubMed: 21607190. PMC3100113 21607190

[36] Leyden JJ, Del Rosso JQ, Webster GF (2007) Clinical considerations in the treatment of acne vulgaris and other inflammatory skin disorders: focus on antibiotic resistance. Cutis 79: 9-25. PubMed: 17649854. 17649854

[37] Crawford WW, Crawford IP, Stoughton RB, Cornell RC (1979) Laboratory induction and clinical occurrence of combined clindamycin and erythromycin resistance in *Corynebacterium acnes* . J Invest Dermatol 72: 187-190. doi:10.1111/1523-1747.ep12676385. PubMed: 429800. 429800 10.1111/1523-1747.ep12676385

[38] Chronnell CM, Ghali LR, Ali RS, Quinn AG, Holland DB et al. (2001) Human beta defensin-1 and -2 expression in human pilosebaceous units: upregulation in acne vulgaris lesions. J Invest Dermatol 117: 1120–1125. doi:10.1046/j.0022-202x.2001.01569.x. PubMed: 11710922. 11710922 10.1046/j.0022-202x.2001.01569.x

[39] Lee DY, Yamasaki K, Rudsil J, Zouboulis CC, Park GT et al. (2008) Sebocytes express functional cathelicidin antimicrobial peptides and can act to kill *propionibacterium acnes* . J Invest Dermatol 128: 1863-1866. doi:10.1038/sj.jid.5701235. PubMed: 18200058. 18200058 10.1038/sj.jid.5701235 PMC2632971

[40] Nizet V (2006) Antimicrobial peptide resistance mechanisms of human bacterial pathogens. Curr Issues Mol Biol 8: 11-26. PubMed: 16450883. 16450883

[41] Yeaman MR, Yount NY (2003) Mechanisms of antimicrobial peptide action and resistance. Pharmacol Rev 55: 27-55. doi:10.1124/pr.55.1.2. PubMed: 12615953. 12615953 10.1124/pr.55.1.2

[42] Schibli DJ, Epand RF, Vogel HJ, Epand RM (2002) Tryptophan-rich antimicrobial peptides: comparative properties and membrane interactions. Biochem Cell Biol 80: 667-677. doi:10.1139/o02-147. PubMed: 12440706. 12440706 10.1139/o02-147

[43] Yang ST, Shin SY, Lee CW, Kim YC, Hahm KS et al. (2003) Selective cytotoxicity following Arg-to-Lys substitution in tritrpticin adopting a unique amphipathic turn structure. FEBS Lett 540: 229-233. doi:10.1016/S0014-5793(03)00266-7. PubMed: 12681513. 12681513 10.1016/s0014-5793(03)00266-7

[44] Strøm MB, Rekdal O, Svendsen JS (2002) Antimicrobial activity of short arginine- and tryptophan-rich peptides. J Pept Sci 8: 431-437. doi:10.1002/psc.398. PubMed: 12212806. 12212806 10.1002/psc.398

[45] Park Y, Lee DG, Jang SH, Woo ER, Jeong HG et al. (2003) A Leu-Lys-rich antimicrobial peptide: activity and mechanism. Biochim Biophys Acta 1645: 172-182. doi:10.1016/S1570-9639(02)00541-1. PubMed: 12573247. 12573247 10.1016/s1570-9639(02)00541-1

[46] Fimland G, Eijsink VG, Nissen-Meyer J (2002) Mutational analysis of the role of tryptophan residues in an antimicrobial peptide. Biochemistry 41: 9508-9515. doi:10.1021/bi025856q. PubMed: 12135373. 12135373 10.1021/bi025856q

[47] Yau WM, Wimley WC, Gawrisch K, White SH (1998) The preference of tryptophan for membrane interfaces. Biochemistry 37: 14713-14718. doi:10.1021/bi980809c. PubMed: 9778346. 9778346 10.1021/bi980809c

[48] Asthana N, Yadav SP, Ghosh JK (2004) Dissection of antibacterial and toxic activity of melittin: a leucine zipper motif plays a crucial role in determining its hemolytic activity but not antibacterial activity. J Biol Chem 279: 55042-55050. doi:10.1074/jbc.M408881200. PubMed: 15475354. 15475354 10.1074/jbc.M408881200

[49] Kondejewski LH, Jelokhani-Niaraki M, Farmer SW, Lix B, Kay CM et al. (1999) Dissociation of antimicrobial and hemolytic activities in cyclic peptide diastereomers by systematic alterations in amphipathicity. J Biol Chem 7: 13181-13192. PubMed: 10224074. 10.1074/jbc.274.19.13181 10224074

[50] Wang Y, Agerberth B, Löthgren A, Almstedt A, Johansson J (1998) Apolipoprotein A-I binds and inhibits the human antibacterial/cytotoxic peptide LL-37. J Biol Chem 273: 33115-33118. doi:10.1074/jbc.273.50.33115. PubMed: 9837875. 9837875 10.1074/jbc.273.50.33115

[51] Barańska-Rybak W, Sonesson A, Nowicki R, Schmidtchen A (2006) Glycosaminoglycans inhibit the antibacterial activity of LL-37 in biological fluids. J Antimicrob Chemother 57: 260-265. doi:10.1093/jac/dki460. PubMed: 16387752. 16387752 10.1093/jac/dki460

[52] Jeremy AH, Holland DB, Roberts SG, Thomson KF, Cunliffe WJ (2003) Inflammatory events are involved in acne lesion initiation. J Invest Dermatol 121: 20–27. doi:10.1046/j.1523-1747.2003.12321.x. PubMed: 12839559. 12839559 10.1046/j.1523-1747.2003.12321.x

